# Metachronous renal cell carcinoma with metastasis to the urinary bladder, and distant organs, 28 years after radical nephrectomy: a case report

**DOI:** 10.1186/s12894-019-0521-1

**Published:** 2019-12-27

**Authors:** Mustufa Babar, Saad Hamdani, Corinne Liu, Jogarao Vedula, David S. Schnapp

**Affiliations:** 1 DSS Urology, Queens Village, New York, USA; 2 Department of Radiology, Memorial Sloan Kettering Cancer Center, New York, USA; 3 Department of Pathology, NYU Winthrop Hospital, Mineola, New York, USA

**Keywords:** Renal cell carcinoma, Bladder metastasis, Nephrectomy

## Abstract

**Background:**

Metachronous renal cell carcinoma after radical nephrectomy is extremely rare. Renal cell carcinoma commonly metastasizes to distant organs. However, metastasis to the urinary bladder is very uncommon.

**Case presentation:**

Herein, we report a case of metachronous renal cell carcinoma with metastasis to the urinary bladder, left acetabulum, left rib, lungs, thyroid, right renal vein and inferior vena cava. The patient had undergone a left-sided radical nephrectomy 28 years ago. The pathological diagnosis of a fragment of the bladder tumor was consistent with Fuhrman grade 2 clear cell renal cell carcinoma.

**Conclusions:**

Although metachronous renal cell carcinoma after radical nephrectomy is rare, active surveillance should be still considered. Renal cell carcinoma has shown to unusually metastasize to the urinary bladder, a rarely reported organ of metastasis. Treatment options, such as immunotherapy, are available to patients with such metastasis and long-term survivorship can be achieved.

## Background

As the third most common urological cancer and with newly reported cases arising each year due to increased usage of imaging procedures, renal cell carcinoma (RCC) proves to be a relevant adult malignancy [1]. Although extremely rare, RCC has the ability to undergo metachronous metastasis many years after radical nephrectomy. Furthermore, RCC frequently metastasizes to distant organs. However, metastasis to the urinary bladder is extremely rare with less than 40 reported cases in literature [2]. We present a case of metachronous RCC with metastasis to the bladder, as well as distant organs, 28 years after radical nephrectomy.

## Case presentation

A 79-year-old man, who had a left-sided radical nephrectomy 28 years ago as a result of renal cell carcinoma, is presented with urinary retention for six months. Renal ultrasound revealed the right kidney to be 13.6 cm, normal echogenicity without hydronephrosis, and a mildly distended bladder. Urodynamic testing indicated obstruction.

**Fig. 1:**
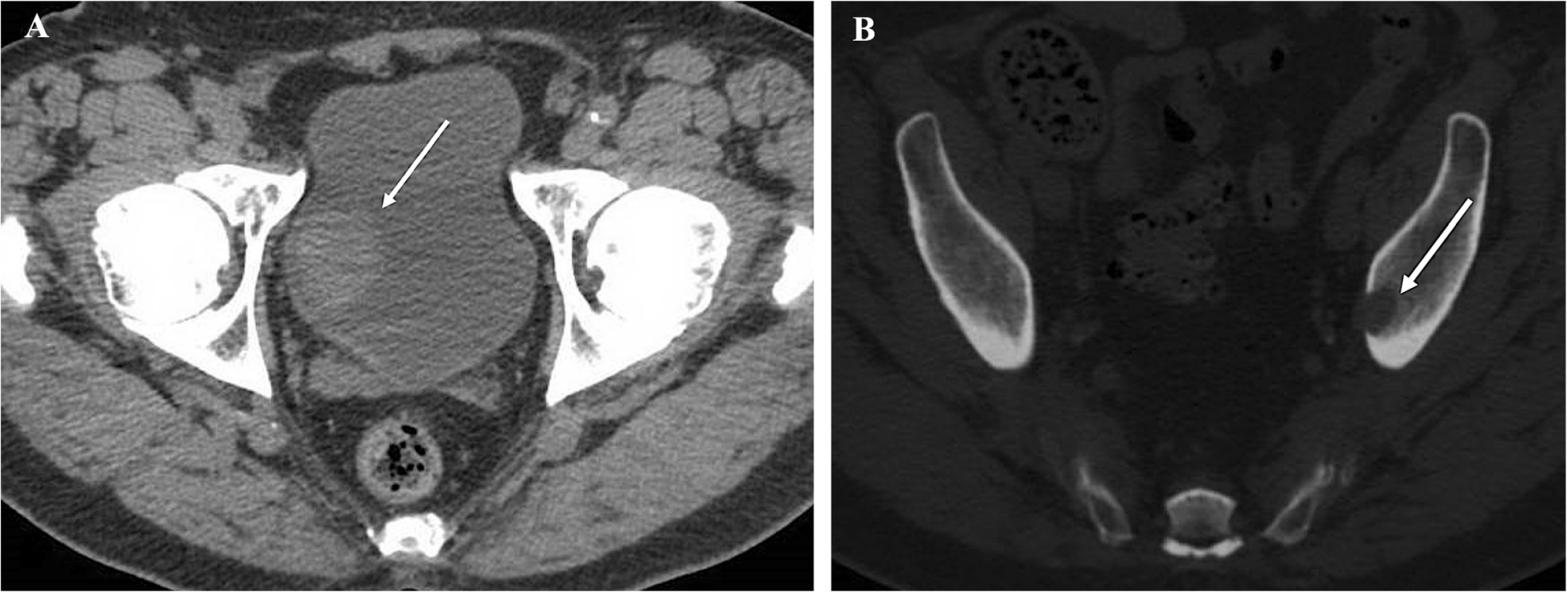
**a** Non-contrast CT abdomen and pelvis demonstrates a hyperattenuating intraluminal bladder mass (white arrow). **b** Non-contrast CT abdomen and pelvis reveal a lytic lesion with cortical destruction and extraosseous soft tissue extension in the left ilium (white arrow), consistent with osseous metastasis

The patient returned one month later with gross hematuria. Computed tomography (CT) scan of the abdomen and pelvis revealed an intraluminal 3.7*3.2 cm mass on the right side of the urinary bladder (Fig. 1a) and a 1.7 cm lytic lesion in the left acetabulum, which was suspicious for metastatic disease (Fig. 1b). Furthermore, the right kidney demonstrated subcentimeter hypodense lesions. Magnetic resonance imaging (MRI) of the visceral pelvis showed an enhancing 2.5 cm lesion in the left superior acetabular region with disruption of the medial cortex that was consistent with metastatic disease.

**Fig. 2:**
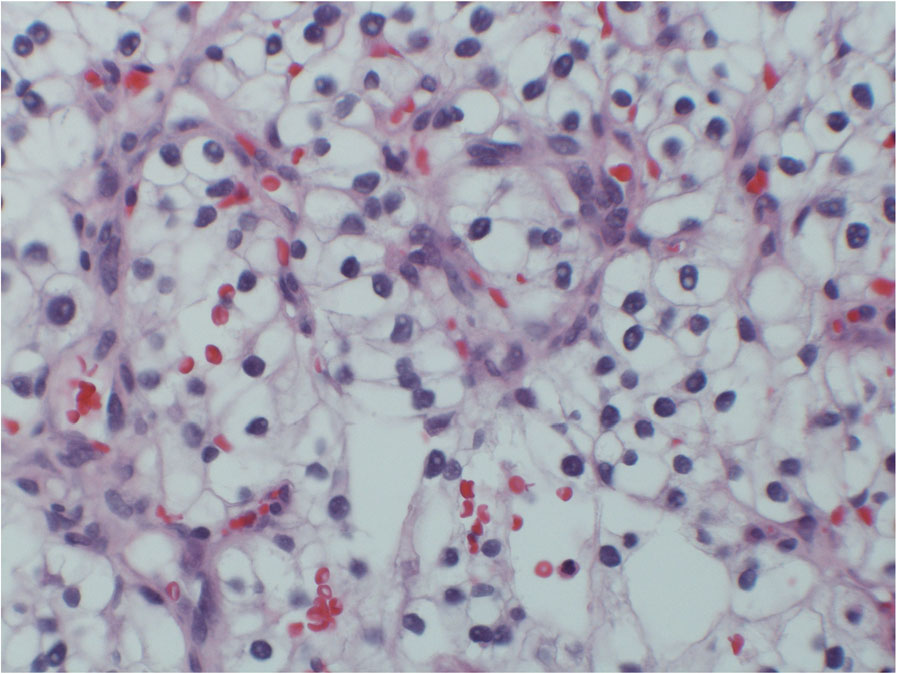
Fuhrman grade 2 renal cell carcinoma metastatic to the urinary bladder. Hematoxylin-eosin stain, reduced from 40x

**Fig. 3:**
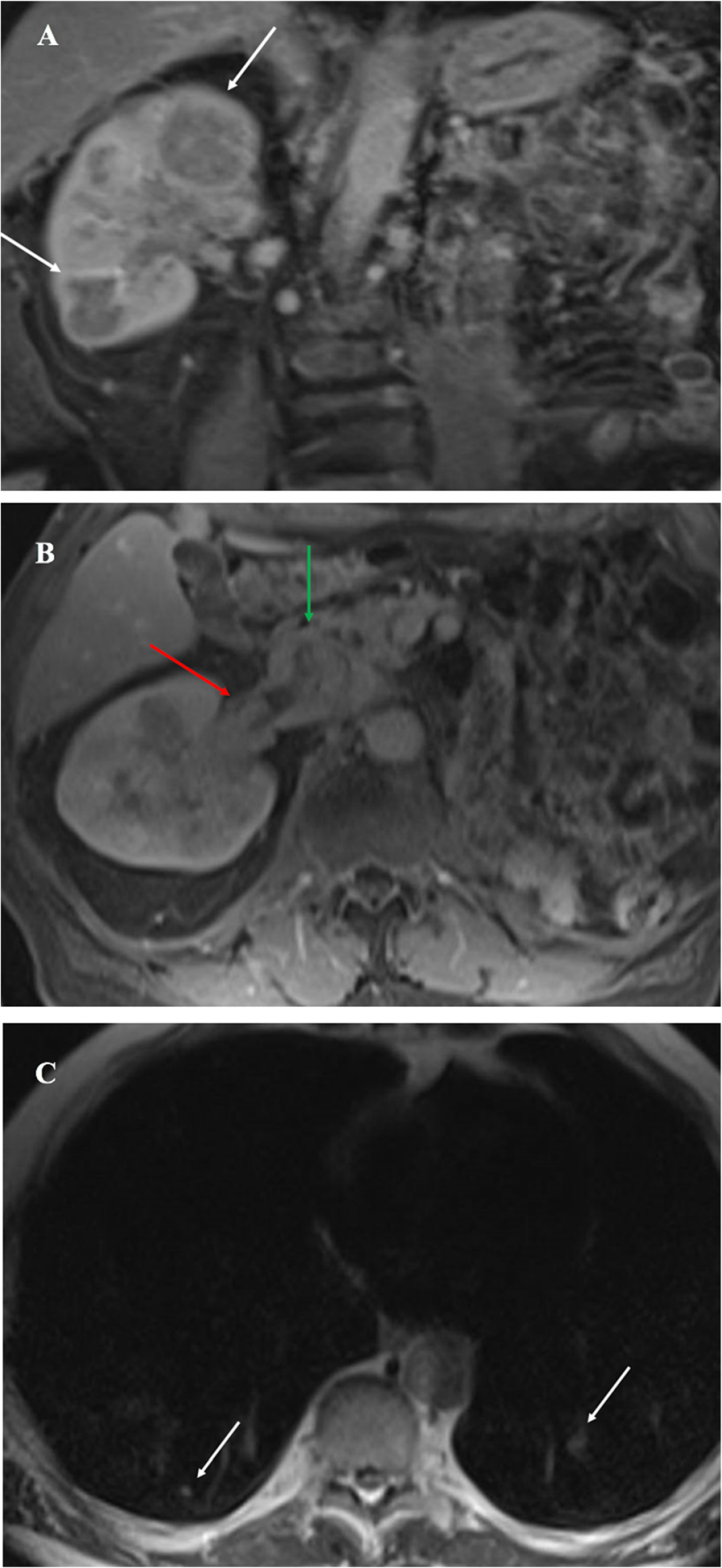
**a** Gadolinium enhanced T1 weighted coronal image of the abdomen demonstrates multiple hypoenhancing masses throughout the right kidney (white arrows). **b** Gadolinium enhanced T1 weighted axial image of the abdomen demonstrates right renal vein (red arrow) and inferior vena cava invasion (green arrow) by tumor. **c** Axial T2 weighted image demonstrates several scattered pulmonary nodules (white arrows)

A transurethral resection of the bladder removed a 4.2*3.5*0.6 cm single fragment of aggregate soft, tan-brown colored bladder tissue. The pathological diagnosis of a fragment of the partially necrotic bladder tumor was consistent with Fuhrman grade 2 clear cell renal cell carcinoma (Fig. 2). Bone scan showed negative findings. However, positron emission tomography computed tomography scan with fluorodeoxyglucose (PET-CT FDG) of the skull base to thighs revealed scattered hypermetabolic lytic osseous lesions in the left acetabulum, a lytic lesion in the 1st left rib, a hypermetabolic 4.4 cm right para-aortic retroperitoneal lesion, numerous subcentimeter scattered lungs nodules, and a 17 mm right thyroid nodule. Furthermore, an MRI of the abdomen revealed multiple hypoenhancing masses in the right kidney which were suspicious for renal neoplasm associated with metastatic disease (Fig. 3a). The MRI also showed a tumor thrombus in the right renal vein and inferior vena cava (Fig. 3b), and pulmonary nodules (Fig. 3c).

At first, the patient decided not to undergo any systemic treatment plan as he desired to pursue the situation through a holistic approach. A few weeks later, however, the patient agreed to start chemotherapy. He was given sunitinib 12.5 mg which was later increased to 25 mg. However, he was unable to tolerate the increased sunitinib dosage due to gross hematuria. Consequently, the patient was given nivolumab through a Port-a-Cath and has remained in stable condition for over two years.

## Discussion and conclusions

Metachronous RCC after radical nephrectomy is extremely rare and reported to occur at 1.2% [3]. Although metachronous RCC occurrence is typically 5 years post nephrectomy [4], rare cases show metachronous RCC occurrence after 24 years [5]. Metachronous RCC requires patients to receive active surveillance as an independent viable option based on personal and financial grounds [4]. Several surveillance protocols, such as routine imaging, physical examination, and laboratory testing, have been suggested as surveillance options barring aggressiveness and staging of the tumor [6]. Furthermore, it is recommended to remain under surveillance 5–10 years post nephrectomy [4]. However, our case suggests that surveillance for life may be necessary.

RCC can metastasize to many distant organs, with lungs, bones, liver and distant lymph nodes being the most common. These common sites of metastasis can be monitored and effectively treated with resection [4]. However, RCC metastasis to the bladder is extremely rare, accounting for less than 2 % of all bladder tumors [7]. Consequently, bladder metastasis is more challenging to treat due to its irregularity. Bladder metastasis frequently presents itself with gross hematuria. Although most patients with RCC metastasis to the bladder die within the first year of diagnosis, long-term survivorship of more than six years have been reported [8, 9]. The mechanism of RCC metastasis to the bladder remains a subject of discussion but several theories have been proposed.

One theory suggests retrograde venous embolism from renal venous drainage through a tumor thrombus [8–10]. More specifically, Abeshouse states that a tumor thrombus found in the left renal vein, as opposed to the right renal vein, is responsible for RCC metastasis to pelvic organs since the left renal vein is the central network of venous circulation to adjacent organs [9, 10]. In our case, the patient had a tumor thrombus in the right renal vein as well as a prior left-sided radical nephrectomy. As a result, for our case, the mechanism of retrograde venous embolism through the left renal vein is unlikely to be responsible for the bladder metastasis observed.

Another theory of RCC metastasis to the bladder suggests metastasis to occur through the lymphatic system, which involves the penetration and embolization of tumor cells through vascular lymphatic vessels [8]. However, lymphatic invasion and an interconnected vascular network between the kidneys and the bladder is not observed [9]. Therefore, RCC metastasis to the bladder through the lymphatic system is an unlikely route.

Raviv et al. used the term “drop metastases” to describe a fascinating theory of RCC metastasis to the bladder by direct seed implantation of cancer cells through the urinary tract [8, 9]. This route of metastasis is proposed due to the presence of tumor cells in the urine of patients with RCC metastasis to the bladder [8]. However, the likelihood of metastasis to the urinary tract was found to be low in these patients which makes this route questionable [9]. In addition, because our patient had systemic metastasis to distant organs, “drop metastases” may not be the suggestable metastatic route in our case.

Another theory of tumor metastasis, known as hematogenous metastasis, involves tumor cells penetrating blood vessels and invading different organs through the general circulation [11]. Since our patient had multiple sites of metastasis, including the bladder, bones, lungs, thyroid, and veins, our case suggests hematogenous metastasis to be responsible for RCC metastasis to the bladder and distant organs. The tumor thrombus observed in the patient’s right renal vein may have spread into his inferior vena cava before extending to the heart and lungs and eventually diffusing to other parts of the body through the systemic circulation [12]. However, since there are many interconnections between these theories of metastasis, several of these routes may be responsible for the unique metastasis observed.

Treatment for RCC metastasis is offered on a case by case basis. For bladder lesions as a result of RCC metastasis, transurethral resection or partial cystectomy is recommended [8]. For metastasis to other organs, systemic treatment options, such as chemotherapy and radiation therapy, should be offered. A chemotherapy option that is found to be effective in managing metastatic and metachronous RCC uses target treatment agents such as sunitinib and sorafenib. These agents provide a novel approach in managing RCC by targeting vascular endothelial and platelet-derived growth factors [11]. Furthermore, immunotherapy, involving nivolumab or IL-2 cytokines, is also found to be effective in managing RCC.

Metachronous RCC after radical nephrectomy is probable, although rare, making active surveillance an important option. In addition, RCC has shown to unusually metastasize to the urinary bladder, a rarely reported organ of metastasis. Although there is no general accepted theory, several theories have been proposed to account for the unique route of metastasis to the bladder. Treatment options are available to patients with such metastasis and long-term survivorship can be achieved. Our patient is alive for more than two years after metachronous RCC with metastasis to the bladder, and other organs, under immunotherapy involving nivolumab.

## Data Availability

Not applicable.

## Abbreviations

CT: Computed tomography
MRI: Magnetic resonance imaging
PET-CT FDG: Positron emission tomography computed tomography scan with fluorodeoxyglucose
RCC: Renal cell carcinoma

## References

[1] Chow W-H, Devesa SS, Warren JL (1999). Rising incidence of renal cell Cancer in the United States. JAMA.

[2] Zhang M, Wah C, Epstein JI (2014). Metastatic renal cell carcinoma to the urinary bladder: a report of 11 cases. Am J Surg Pathol.

[3] Bani-Hani AH, Leibovich BC, Lohse CM (2005). Associations with contralateral recurrence following nephrectomy for renal cell carcinoma using a cohort of 2,352 patients. J Urol.

[4] Abara E, Chivulescu I, Clerk N (2010). Recurrent renal cell cancer: 10 years or more after nephrectomy. Can Urol Assoc J.

[5] Donaldson JC, Slease RB, DuFour DR (1976). Metastatic renal cell carcinoma 24 years after nephrectomy. JAMA.

[6] Chin AI, Lam JS, Figlin RA (2006). Surveillance strategies for renal cell carcinoma patients following nephrectomy. Rev Urol.

[7] Roberts DI (1978). Secondary neoplasms of the Genito-urinary tract. Br J Urol.

[8] Shiraishi K, Mohri J, Inoue R (2003). Metastatic renal cell carcinoma to the bladder 12 years after radical nephrectomy. Int J Urol.

[9] Raviv Stacy, Eggener Scott E, Williams Dan H, Garnett John E, Pins Michael R, Smith Norm D (2002). Long-term survival after “drop metastases” of renal cell carcinoma to the bladder. Urology.

[10] Abeshouse BS (1956). Metastasis to ureters and urinary bladder from renal carcinoma: report of two cases. J Int Coll Surg.

[11] Doo SW, Kim WB, Kim BK (2013). Metastasis of renal cell carcinoma to the bladder. Korean J Urol.

[12] Brufau BP, Cerqueda CS, Villalba LB (2003). Metastatic renal cell carcinoma: radiologic findings and assessment of response to targeted antiangiogenic therapy by using multidetector CT. RadioGraphics.

